# Supplementary material for: Nanopore-based single molecule sequencing of the D4Z4 array responsible for facioscapulohumeral muscular dystrophy
Source: Sci Rep. 2017 Nov 1;7:14789. doi: 10.1038/s41598-017-13712-6 (PMC5665936; doi:10.1038/s41598-017-13712-6)
Supplement: Supplementary file 1 — Supplemental Data [file 41598_2017_13712_MOESM1_ESM.pdf]

Nanopore-based single molecule sequencing of the D4Z4 array responsible for facioscapulohumeral muscular dystrophy

Satomi Mitsuhashi[1,2], So Nakagawa[1,3], Mahoko Takahashi Ueda[1,3], Tadashi Imanishi[1], Martin C Frith[4-6], Hiroaki Mitsuhashi[7]

1. Biomedical Informatics Laboratory, Department of Molecular Life Science, Tokai University School of Medicine, Isehara, Kanagawa 259-1193, Japan
2. Department of Human Genetics, Yokohama City University Graduate School of Medicine, Yokohama, Kanagawa 236-0004, Japan
3. Micro/Nano Technology Center, Tokai University, Hiratsuka, Kanagawa, 259-1291, Japan
4. Artificial Intelligence Research Center, National Institute of Advanced Industrial Science and Technology (AIST), Tokyo, 135-0064, Japan
5. Graduate School of Frontier Sciences, University of Tokyo, Chiba, 277-8562, Japan

6. Computational Bio Big-Data Open Innovation Laboratory (CBBD-OIL), National Institute of Advanced Industrial Science and Technology (AIST), Tokyo, 169-8555, Japan
7. Department of Applied Biochemistry, School of Engineering, Tokai University, Hiratsuka, Kanagawa, 259-1291, Japan

Corresponding author

Satomi Mitsuhashi

Department of Human Genetics Yokohama City University Graduate School of Medicine, Yokohama, Kanagawa 236-0004, Japan

Tel. +81-45-787-2606

Fax. +81-45-786-5219

E-mail: satomits@yokohama-cu.ac.jp

Supplemental Figure 1

The last D4Z4 repeat

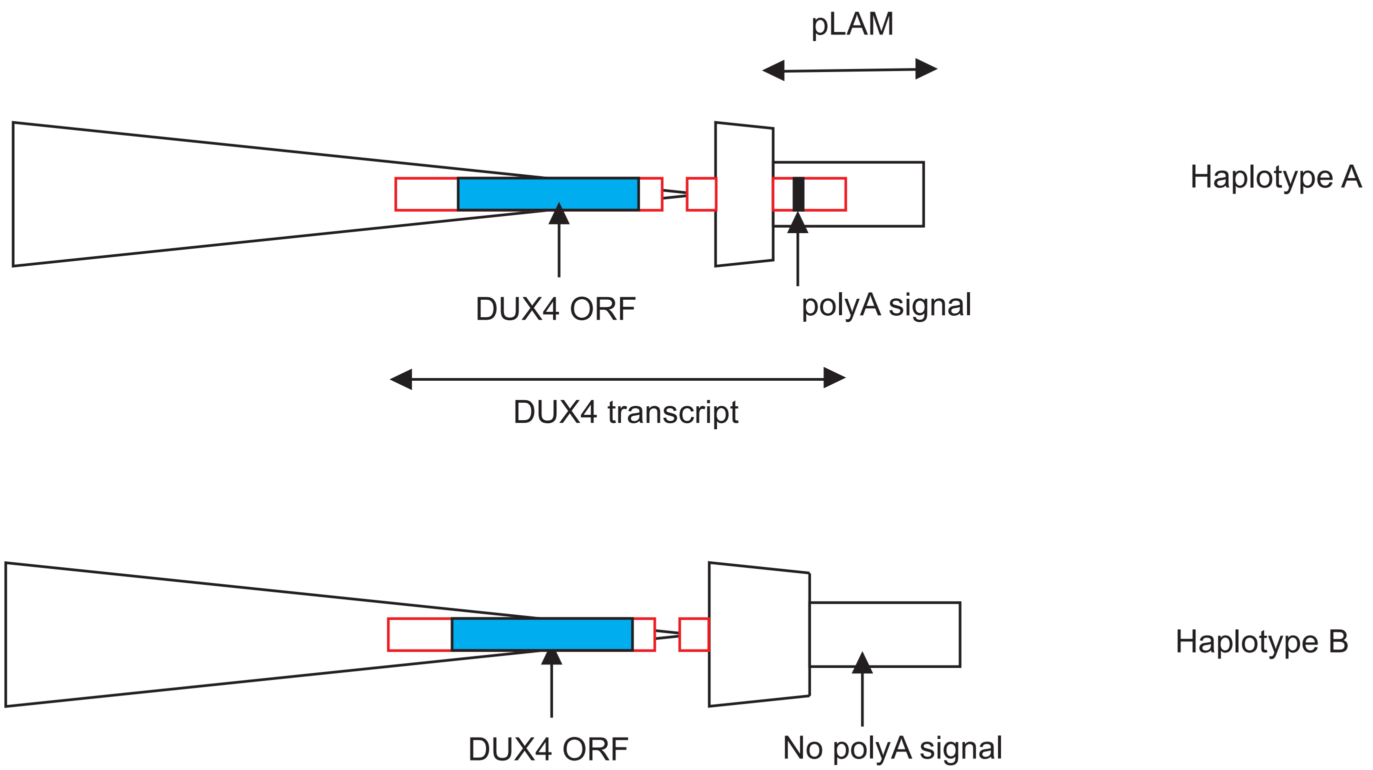

Supplemental figure 2

a

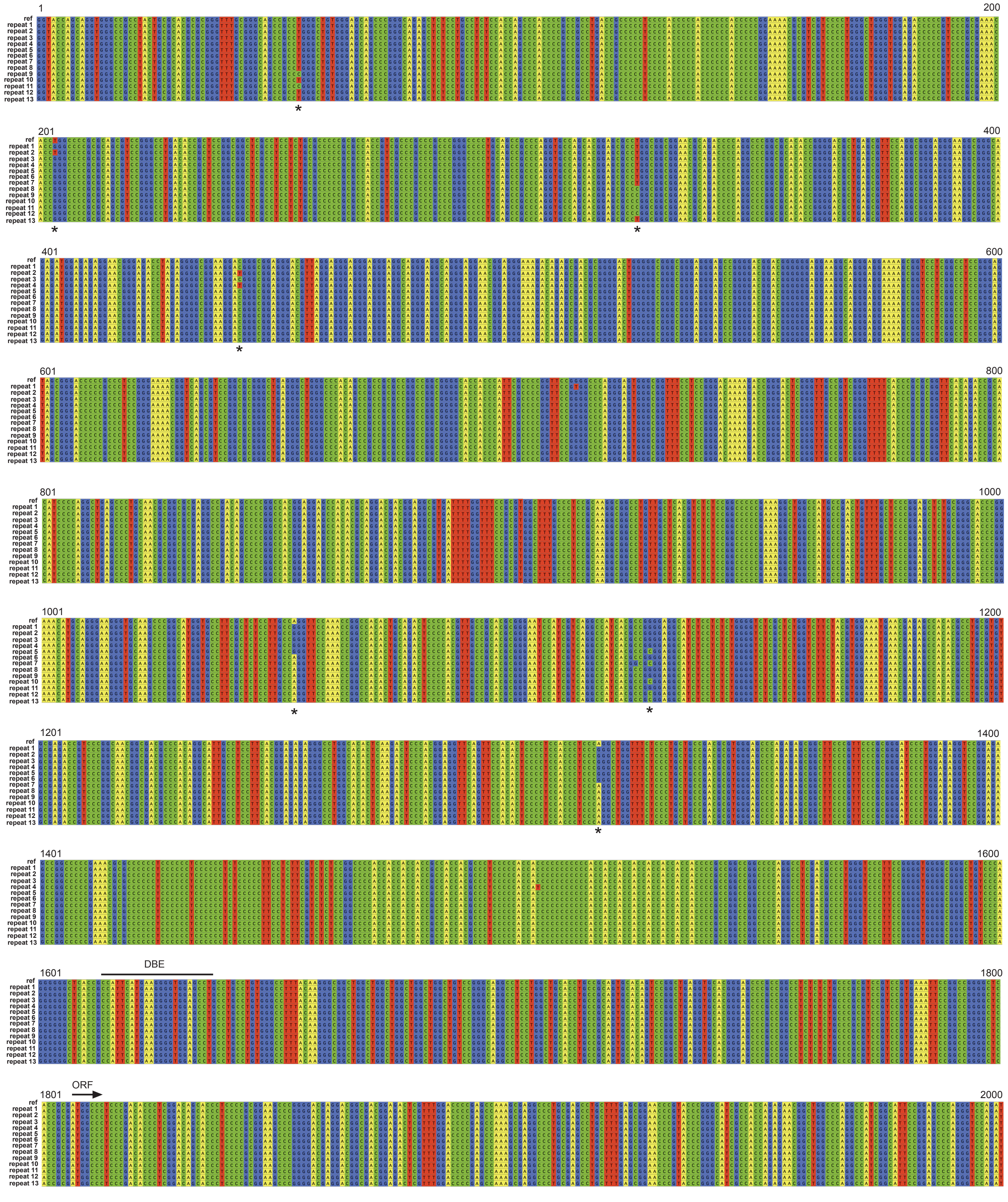

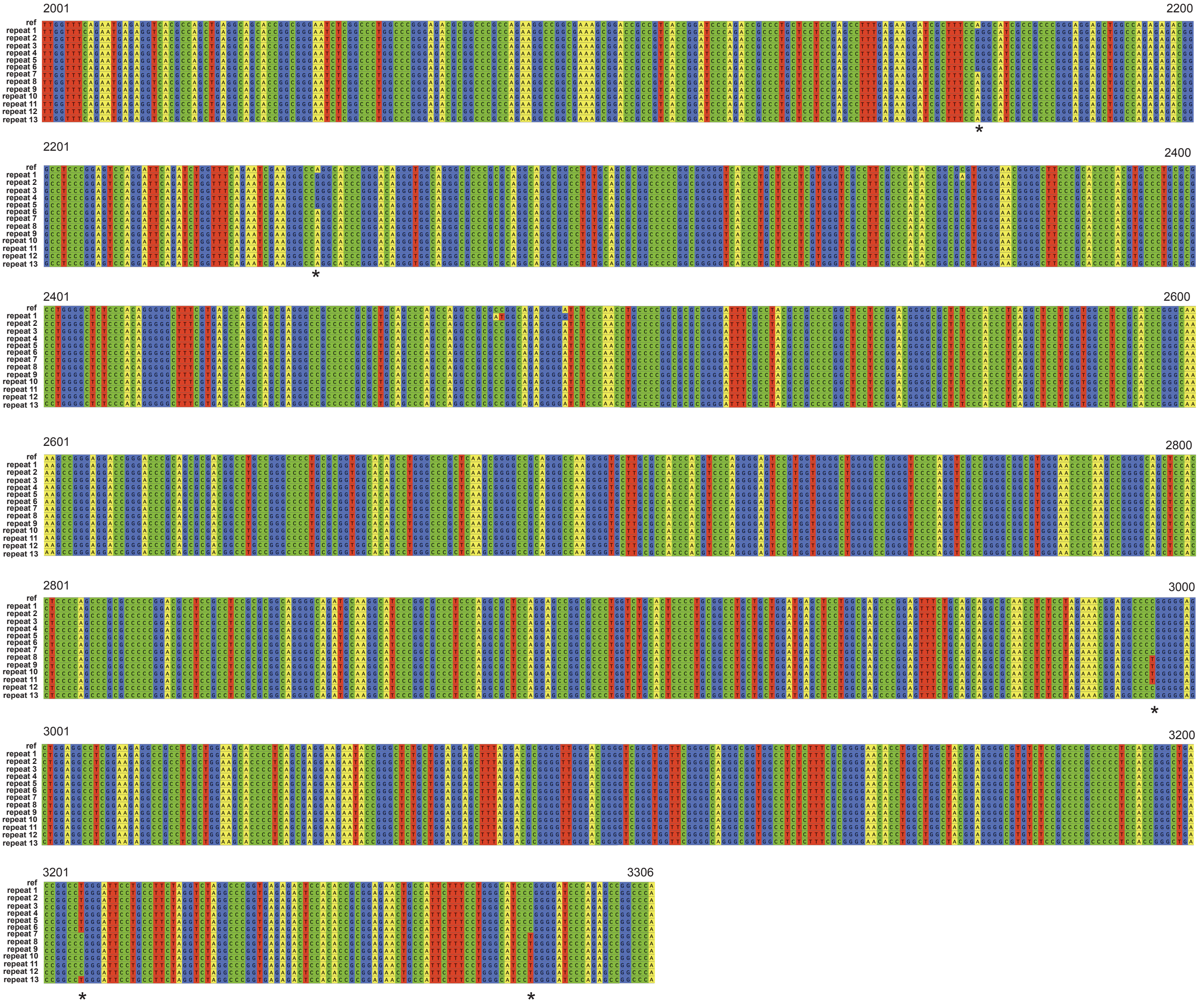

b

		MinION			
		A	C	G	T
ref	A		0	25	0
	C	0		13	13
	G	0	1		5
	T	2	15	1	

Supplemental Figure 3

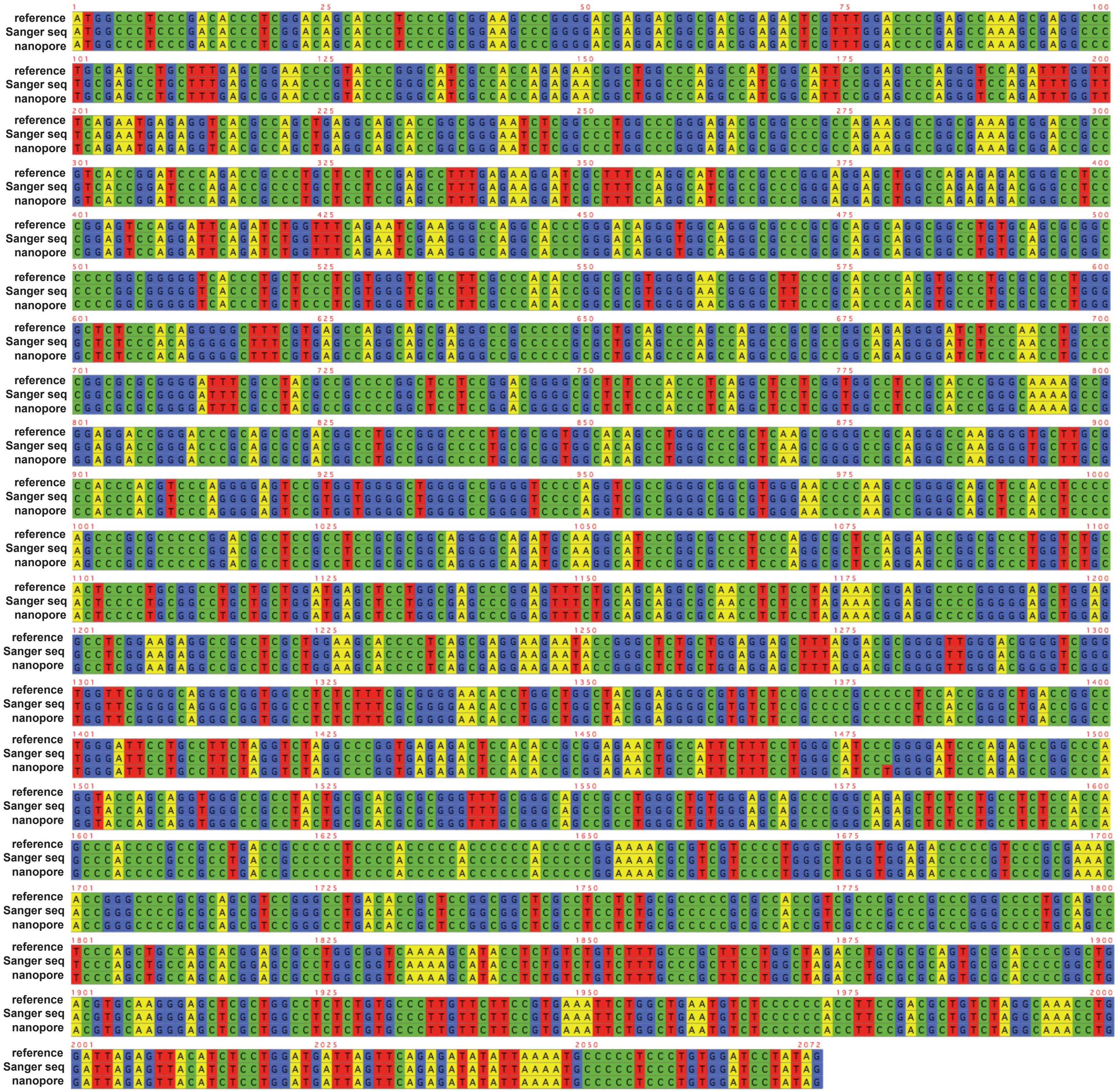

Supplemental Figure 4

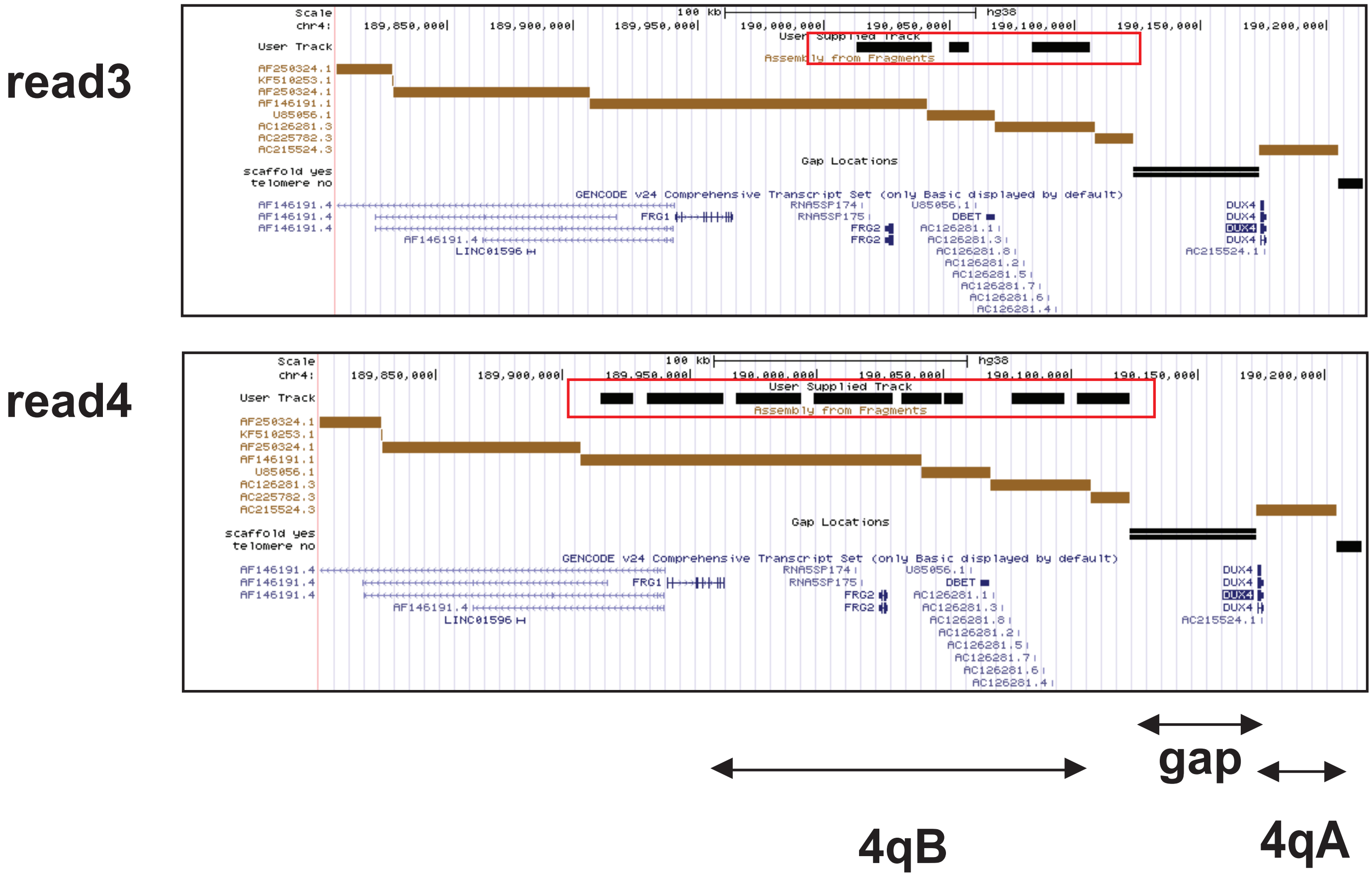

Supplemental Figure 5

a

The last D4Z4 - 4qB
read3

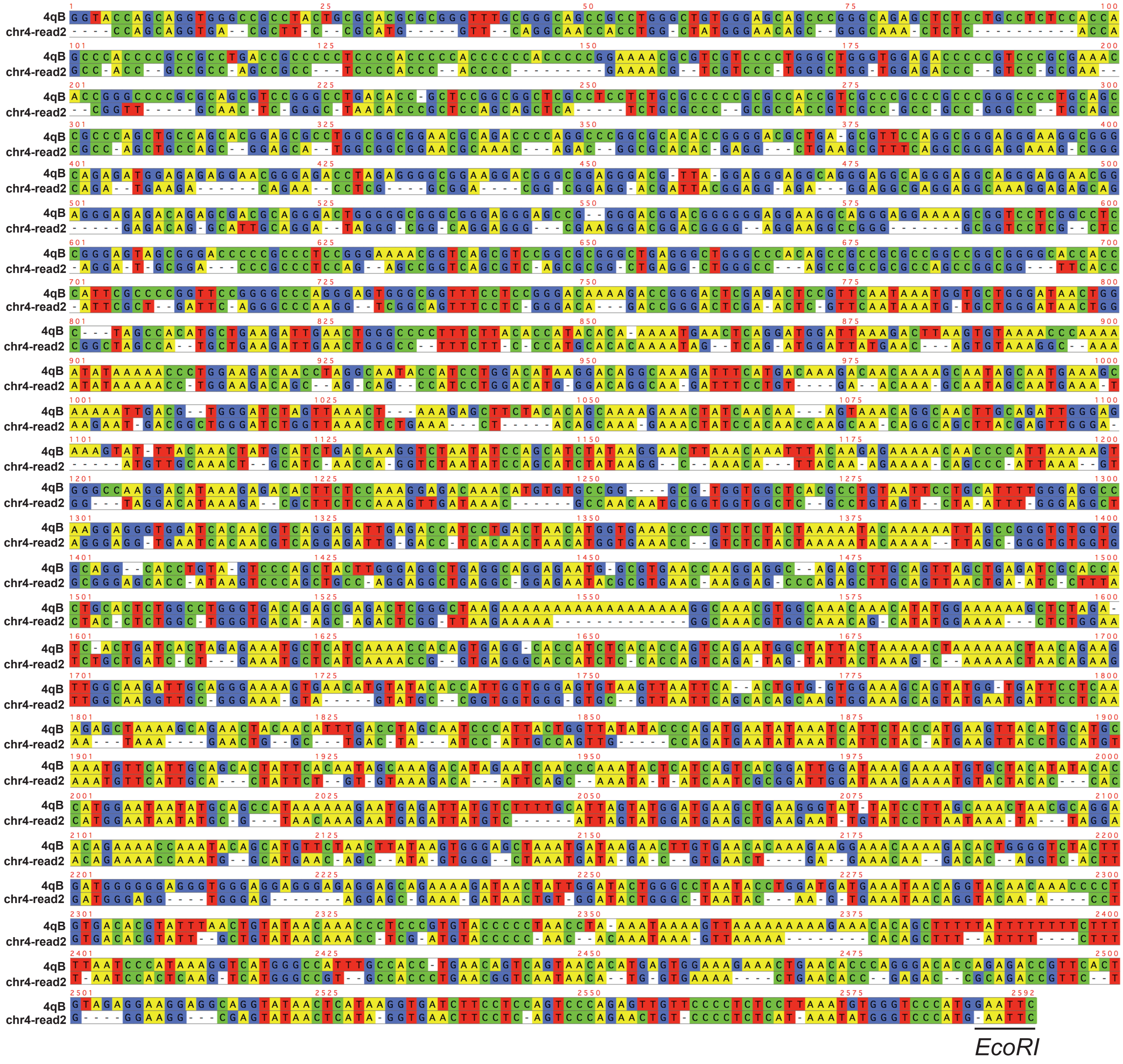

b

The last D4Z4 - 4qB

read4

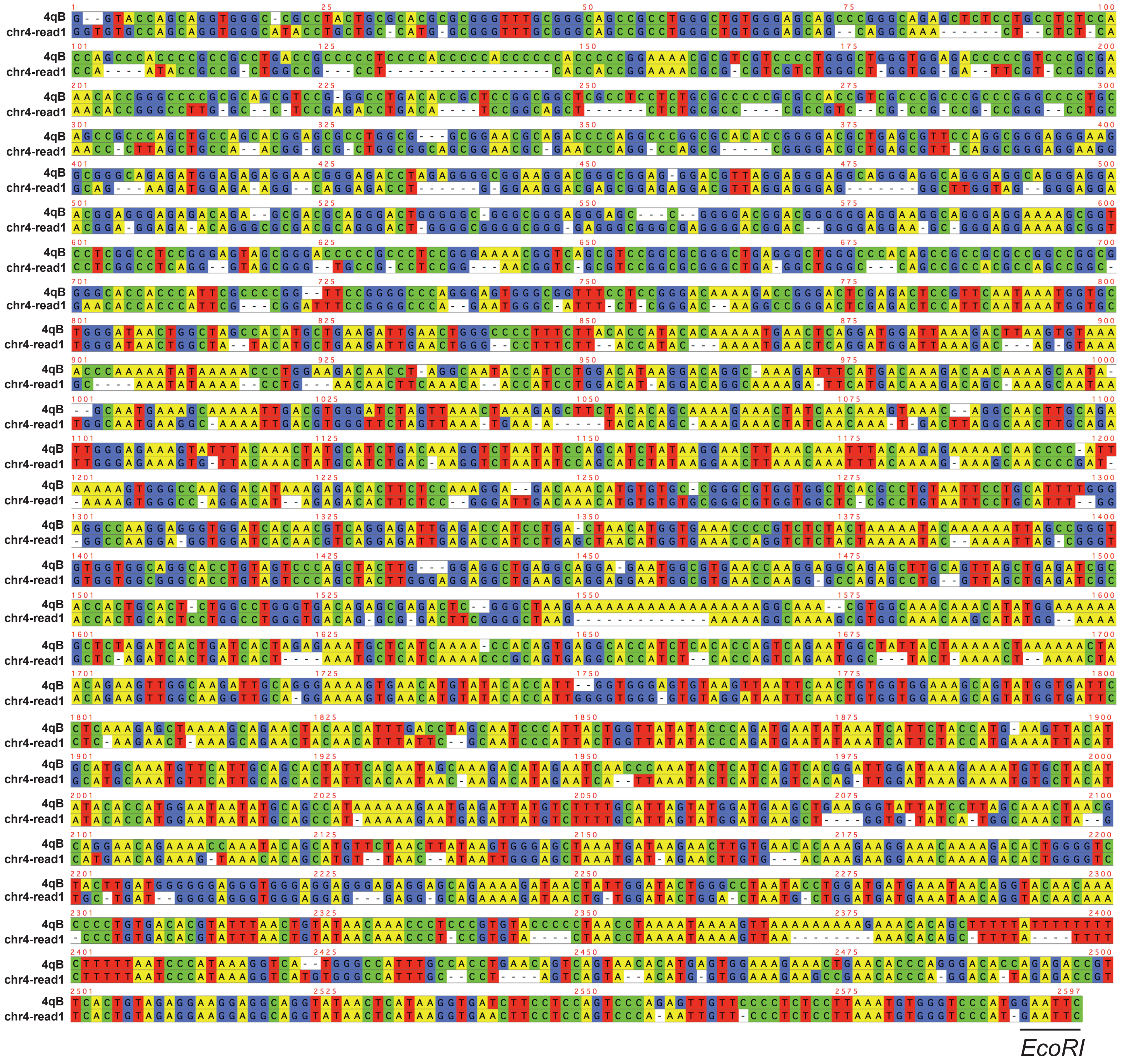

	Statistics
Number of reads	128,171
Total bases	971,130,587
Average read length	7,577

Supplemental Table 1. Statistics of the MinION sequencing

Supplemental Table 1.

We obtained 128,171 reads from the single MinION run using the R9.4 flow-cell.

	Number of base	(%)
A	7907	15.9
T	7295	14.6
C	18074	36.2
G	16601	33.3
total	49877	

Supplemental Table 2. Composition of the sequenced D4Z4 fragment

Supplemental Table 2.

Summary of the base content of the sequenced region. The D4Z4 array is a GC-rich sequence.

	Read ID	Length of the read	Position of the first repeat starts	Position of the last repeat ends	Length of the D4Z4 repeats	Read mapped to	# of repeat estimated from single D4Z4 alingment
read1	779f5ab0-c45c-4c80-9950-8848b24d70a4_Basecall_1D_template	93,975	13,956	67,645	53,689	chr10	20
read2	05e99c44-b96e-4ebe-a83c-f3eed6c27a39_Basecall_1D_template	158,136	68,858	123,650	54,792	chr10	20
read3	915c2d77-8227-4803-957c-a3c8d3ee4981_Basecall_1D_template	97,106	42,093	85,838	43,745	chr4	17
read4	457c6e90-cc88-48d4-91ec-f3fabbf321ec_Basecall_1D_template	201,153	127,619	174,409	46,790	chr4	17

Supplemental Table 3. Statistics of the 4 reads containing chr4 and chr10 D4Z4.

Supplemental Table 3.

From the rel4 dataset, we obtained 4 reads mapped to chr4 and chr10 that contain the whole D4Z4 array. The statistics of the reads are described. The original read ID are also shown.

Figure legend

Supplemental Figure 1

The scheme shows haplotype 4qA and 4qB. There is no homology between the pLAM regions and 4qB. 4qB lacks polyA signaling thus considered to be benign.

Supplemental Figure 2

(a) Alignment of the nanopore-derived consensus sequence to the 13 D4Z4 repeats reference (CT476828.7). Asterisks show the recurrent errors across the repeats. DBE: D4Z4-binding element. The upper lane “ref” shows the single reference D4Z4 sequence. Note that CT476828.7 contains completely identical D4Z4 sequence in each repeat. (b) In total, 75 bases were different from the whole D4Z4 reference sequence. Errors between purines or between pyrimidines are highlighted blue.

Supplemental Figure 3

Comparison of the reference, Sanger sequencing, and nanopore sequencing results of the DUX4 ORF in the last D4Z4 repeat. These sequences are completely matched.

Supplemental Figure 4

The alignment of the two reads obtained by whole human genome nanopore sequencing to the human reference GRCh38. Two reads are viewed using UCSC genome browser and marked by red-circles. Note that there is a false gap between the D4Z4 array with 4qB haplotype and the last D4Z4 repeat with 4qA haplotype sequence. The reads are only aligned to the 4qB region.

Supplemental Figure 5

The 2 reads mapped to chr4 are aligned to the reference sequence with D4Z4 repeat with 4qB haplotype (AC225782.3). The last D4Z4 is shown. The upper sequence is the reference and the lower sequence is the sequence from 2 reads, read3 (a) and read4 (b).
